# Anderson localization of flexural waves in disordered elastic beams

**DOI:** 10.1038/s41598-019-39623-2

**Published:** 2019-03-05

**Authors:** Jesús Calleja Ángel, José Concepción Torres Guzmán, Alfredo Díaz de Anda

**Affiliations:** 1 Instituto de Física, Benemérita Universidad Autónoma de Puebla, P.O. Box J-48, 72570 Puebla, Pue. Mexico; 2 Universidad Politécnica del Estado de Morelos, Jiutepec, Morelos 62550 Mexico; 3 Tecnológico de Monterrey, Campus Estado de Mexico, Atizapán de Zaragoza, 52926 Estado de México Mexico

## Abstract

We study, both experimentally and numerically, the Anderson localization phenomenon in flexural waves of a disordered elastic beam, which consists of a beam with randomly spaced notches. We found that the effect of the disorder on the system is stronger above a crossover frequency *f*_*c*_ than below it. For a chosen value of disorder, we show that above *f*_*c*_ the normal-mode wave functions are localized as occurs in disordered solids, while below *f*_*c*_ the wave functions are partially and fully extended, but their dependence on the frequency is not governed by a monotonous relationship, as occurs in other classical and quantum systems. These findings were corroborated with the calculation of the participation ratio, the localization length and a level statistics. In particular, the nearest spacing distribution is obtained and analyzed with a suitable phenomenological expression, related to the level repulsion.

## Introduction

In condensed-matter physics, a perfect lattice has a level energy spectrum in the form of bands with their corresponding wave functions extended. However, if the system presents random imperfections, wave functions can be localized. This phenomenon is known as Anderson localization named after the work of Anderson^1^ and is one of the most important subjects in condensed matter physics since it is essential to understand the transport properties of materials. It has relevance not only in solid-state studies^2–7^, but also in optics^8–13^, cold atomic gases^14^, microwaves^15–18^, and acoustics^19–24^. Random imperfections in a system are represented, for example, by the presence of strange atoms in an otherwise perfect structure or when there are unit cells of different size, which causes wave functions to be localized and therefore affects the conductivity of the system. Thus, band theory and the theory of the Anderson localization allow us to understand why some materials conduct electricity and why others do not. Experimental studies of the Anderson localization are mainly related to the transmission coefficient or correlations while measurements of wave amplitudes are rather scarce^16,17,25^. In this aspect, classical wave-like phenomena like elastic vibrating systems, offer an unique advantage over quantum systems: in elastic experiments one can measure the wave amplitudes at each spatial point. This allows us to understand the Anderson localization phenomenon in a deeper way.

Recently, the Anderson localization has been studied using elastic vibrating rods^26,27^, in particular torsional wave amplitudes were measured in order to obtain the localization length, as a function of the frequency. Other measures like nearest-neighbor spacing and the inverse participation ratio were obtained from the experiment. Besides torsional waves, one-dimensional elastic systems also exhibit another types of oscillations like compressional and flexural vibrations. The first type is described, as well as the torsional oscillations, by a wave equation, while the flexural type, is better described by fourth-order differential equations instead, according to the Timoshenko beam theory (TBT)^28^. They result from the coupling of two second order differential Navier equations and as a consequence of this coupling, flexural oscillations has several characteristics not observed in compressional or torsional vibrations, one of them is the existence of a crossover frequency, beyond that, vibrations exhibit two wavelengths^29,30^ instead of one, as occurs for frequencies below the crossover one. Since the localization length has a strong dependence on the frequency in both classical and quantum cases, a natural question that arises is how the Anderson localization phenomenon is affected by the crossover frequency.

## Results

**Figure 1:**
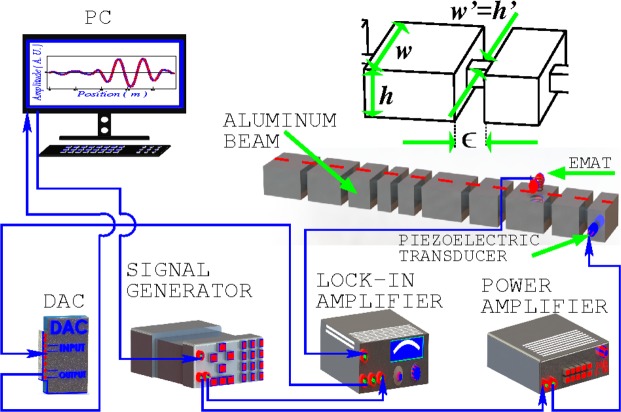
Structured beam used in the experiment to measure the Anderson localization. The number of beams *N*, is 10, all of them having a wide *w* = 2.52 cm and depth *h* = 1.26 cm. The average value of the length of beams *d* is 0.1 m, the length of the system is *L* = 0.95 m and the amount of disorder is Δ = 0.4. The smaller beams have a length *ε* = 3 mm, wide *w*′ = 5.6 mm and depth *h*′ = 5.6 mm. Thus, the corresponding value of coupling parameter is $\eta =0.\bar{2}$. The measured elastic constants are the shear modulus *G* = 27.479 GPa, the Young modulus *E* = 68.821 GPa, and the density *ρ* = 2755.12 kg/m^3^. The experimental setup used to measure flexural frequencies is shown in the lower part of the figure.

In this letter we study flexural vibrations in a disordered beam, such as the one shown in Fig. 1. The system consists of *N* beams of wide *w* and depth *h* with lengths *d*_*i*_, *i* = 1, …, *N*, joined by smaller beams of length $\varepsilon \ll {d}_{i}$ ∀*i*, wide *w*′ = *ηw* and depth *h*′, where the coupling constant *η* is such that 0 < *η* < 1. The total length of the system is *L* = 0.95 m. By taking the family {*d*_*i*_ = *d*(1 − *n*_*i*_Δ)} with *n*_*i*_ an uncorrelated random number in the interval [−1, 1], the set of numbers {*d*_*i*_} are uniformly distributed in the interval [*d*(1 − Δ), *d*(1 + Δ)]. Here *d* = 〈*d*_*i*_〉 is the average of *d*_*i*_ and Δ measures the disorder. Notice that, on average, with this disorder, the total length of the beam does not change.

**Figure 2:**
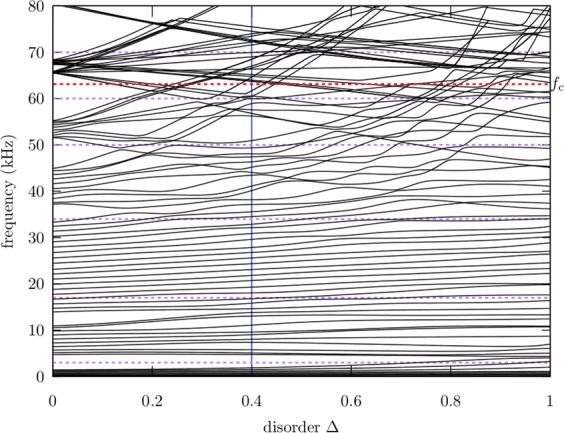
Normal-mode frequencies of a disordered beam, obtained numerically by using TBT, as a function of the disorder Δ. The vertical line lies at Δ = 0.4, the value chosen in the experiment. Note the qualitative change around *f*_c_ = 63.1 kHz indicated by a red horizontal dashed line. The spectrum has been divided in intervals delimited by purple horizontal dashed lines according to Table 1.

In Fig. 2 we show how the spectrum changes with the disorder strength Δ for a fixed realization of *n*_*i*_. One can notice that for ${\rm{\Delta }}\ll 1$ a band spectrum appears; at higher values of Δ, avoided crossings are observed and the structure of bands and gaps disappear. However, one can distinguish two regimes below and above the crossover frequency *f*_*c*_, which is indicated in Fig. 2 by a horizontal dashed line at 63.1 kHz. We remark that this qualitative change is not observed in other elastic systems.

As the value of Δ is increased up to approximately 0.2, the bands and gaps disappear around and above *f*_*c*_, while they remain in the lower part of the spectrum. One can observe that only when the value of Δ is increased up to 0.8, the bands and gaps below *f*_*c*_ indeed disappear and avoided crossings become more evident. Therefore the effect of disorder in the system is stronger above the crossover frequency than below it. To corroborate this hypothesis we have chosen a value of 0.4 for Δ in the experiment and measured the normal-mode frequencies and some of their corresponding wave amplitudes. On the one hand, the degree of localization of the normal-mode wave functions is estimated by two approaches: calculating the inverse participation ratio (IPR) and the localization length *ξ*. On the other hand, the spectrum also provides the nearest-neighbor spacing distribution, which can be described by a frequency-dependent parameter *α* when a distribution, proposed and used to study the kicked rotor model in ref.^31^ is fitted.

### Experimental results

Before the experimental and numerical results are presented, we will mention some remarks about the so called independent rod model, successfully used in dealing with elastic localized states of the Wannier-Stark ladders^32^, which states that introducing disorder in {*d*_*i*_} is a way to simulate diagonal disorder in a quantum mechanical one-dimensional tight-binding Hamiltonian, where the coupling *η* between nearest neighbors is a constant^33^.

According to the independent beam model, a system of beams, like the ones shown in Fig. 1, in which the coupling parameter *η* is small $\eta \ll 1$, the small beams of length *d*_*i*_ behave almost independent of each other. For flexural vibrations and at low frequencies, the Bernoulli-Euler formula holds and a resonant frequency *f*_*i*_ of the *i*-th independent beam is inversely proportional to the square of its length *d*_*i*_. As the frequency increases, the dependence on *d*_*i*_ becomes more complex, however, it is still well described on the average, by the inverse of the square of the length *d*_*i*_ of the beam^30,34^.

**Figure 3:**
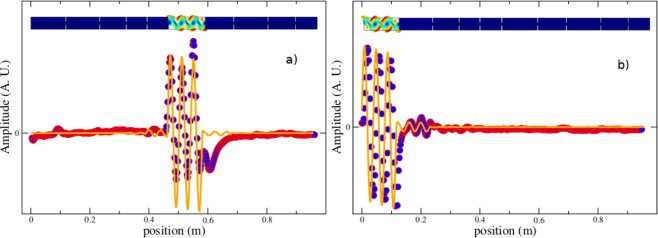
Wave amplitudes as a function of the position along the beam *z*, corresponding to two normal modes of frequency (**a**) *f* = 63.265 kHz and (**b**) *f* = 63.854 kHz, respectively. The continuous line corresponds to a TBT calculation, while filled circles correspond to experimental measurements. Above each plot is shown a three dimensional finite element method (FEM-3D) calculation, where it can be observed the different constituent beams of the system, and the amplitudes of the wave function within each individual beam in a color scale. These calculations were performed using the FEM platform Comsol^53^. The system was modeled with 103, 283 nodes and 309, 849 degrees of freedom (DOF).

When the resonant frequency *f* of the whole system fulfills *f* = *f*_*i*_, the amplitude is maximum at the location of the *i*-th beam. Furthermore, since in general *d*_*j*_ ≠ *d*_*i*_ for *i* ≠ *j*, the neighboring beams of the *i*-th one will be excited only with a smaller amplitude. The states are then localized as shown in Fig. 3, where two wave functions of frequencies 63.265 and 63.854 kHz, respectively, were experimentally measured and in each case, the amplitude is maximum only in one constituent sub-beam. A FEM-3D calculation is also in agreement with the experimental observations, it shows the amplitudes of vibration for every different sub-beam in a color scale. Thus, the amplitude of the wavefunctions with frequencies 63.265 and 63.854 kHz, are maximum only in the sixth and first sub-beam, respectively. Note that these two examples occur at frequencies above *f*_*c*_.

**Figure 4:**
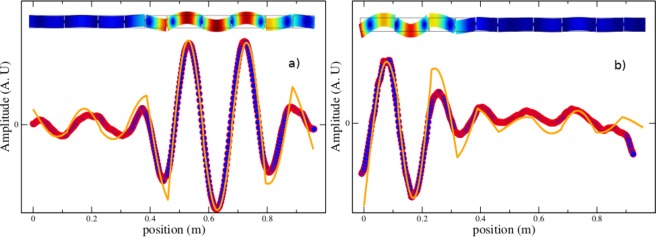
Measured wave amplitudes as a function of the position along the beam *z*, corresponding to two normal modes of frequencies (**a**) *f* = 4.768 kHz and (**b**) *f* = 5.125 kHz, respectively.

When the disorder is very small, on the other hand, all the sub-beams should be excited with a driving frequency *f*, so the localization of the wave amplitude grows and could reach the size of the system, as shown in Fig. 4, where the amplitude of the wave is maximum over almost half the size of the system, i.e., the wave is extended. It is remarkable that these two extreme regimes are indeed observed in the same structured beam. In order to understand how this is possible, we point out that above the crossover frequency *f*_*c*_, the complexity of the relation between the resonant frequencies and *d*_*i*_ grows even more, since doublets appear: a series of resonant frequencies grouped in pairs where the members of each pair are very close in frequency, as shown in refs^30,35^.

The presence of doublets cause an increment in the density of states of the *i*-th independent beam by a factor of two, on the average, compared with the density below *f*_*c*_. Correspondingly, the density of states for a structured beam like the one shown in Fig. 1, also increases above *f*_*c*_ roughly by the same factor. This increment in the average proximity of neighbor frequencies rises the probability of avoided crossings and the Anderson localization to occur when a disorder is introduced in the system through the parameter Δ. Therefore, the effect of introducing a disorder Δ in the system is stronger above *f*_*c*_ than below it. In Fig. 3 we show that the chosen value of Δ = 0.4, is high enough to observe the phenomenon of localization above *f*_*c*_, while below it, the disorder is too small that non localized waves are indeed observed, as shown in Fig. 4.

### Localization measures

**Figure 5:**
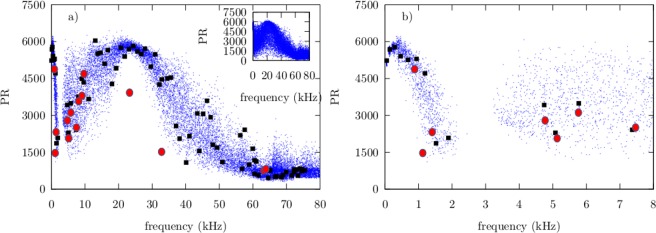
(**a**) Participation ratio PR as a function of frequency for the normal modes of the beam shown in Fig. 1. Filled (black) squares were obtained using TBT, while filled (red) circles were obtained from the experiment. (Blue) dots correspond to an ensemble of 200 beams with Δ = 0.4. Inset: Participation ratio PR as a function of frequency corresponding to the same ensemble of 200 beams but with Δ = 1. (**b**) A zoom at low frequencies.

**Figure 6:**
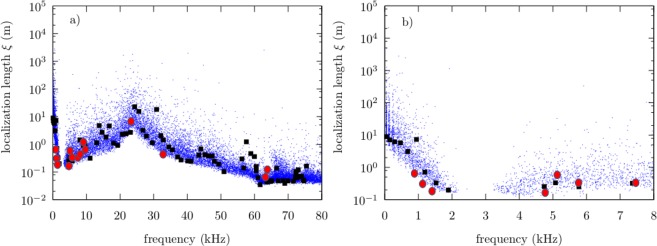
(**a**) Localization length *ξ* as a function of frequency for the normal modes of the beam shown in Fig. 1. Filled (black) squares were obtained using TBT, while filled (red) circles were obtained from the experiment. (Blue) dots correspond to an ensemble of 200 beams with Δ = 0.4. (**b**) Zoom of the localization length *ξ* at low frequencies.

In order to discuss the degree of localization of the normal-mode waves, we have calculated the participation ratio (PR) and the length of localization *ξ* of the normal-mode wave functions of an ensemble of 200 disordered beams with a disorder Δ = 0.4 and using TBT. Here, we find more convenient the PR instead of its reciprocal, the inverse participation ratio (IPR). The PR is compared in Fig. 5 with the ones calculated from the experiment, while the localization length *ξ* is compared in Fig. 6. Notice that low values of both PR and *ξ* are observed above *f*_*c*_ ≈ 63.1 kHz, in agreement with the localized wave functions discussed above, while below *f*_*c*_ both values of PR and *ξ* reveal a rather complex relation as a function of the frequency.

At very low frequencies, the first normal-modes frequencies occur at practically the same values when *f* < 1 kHz and the PR values corresponding to the wave functions suggest that they are extended, independently of the random realization, as can be observed in Fig. 5(b), where a zoom in the regime of low frequencies is shown. Meanwhile, the corresponding *ξ* values show a large localization length, which can exceed the actual size of the system by several orders of magnitude, as observed in the zoom of Fig. 6(b) and corroborate the extended nature of the normal-mode wave functions. This situation is consistent with one where the disorder has no significant effect in the system.

Around the frequency value of *f* = 2 kHz, a residual first band ends and a gap begins, which is consistent with a quasi-periodic spectrum and indicative of a weak effect of the disorder in the system. In the interval 1 to 2 kHz, the normal-mode frequencies begin to variate depending on the random realization and the corresponding PR values decrease, which suggest a localization process. The same trend is observed in the values of *ξ*, which reach low values up to 0.2 m. A close look to the wave functions, allows us to conclude that these functions have significant amplitudes only in several sub beams, while in the rest of them they are substantially reduced. We observe that the end states of the residual band appear to react to the disorder.

A similar situation can be encountered at the beginning of the residual second band, around 3.5 Hz as observed in Figs 5(b) and 6(b). However, in the interval 3.5 to 17 kHz, both the PR and *ξ* values are more disperse, with an average value that tends to grow as the normal-mode frequency does. This observation suggest the occurrence of partial and fully extended wave functions as the frequency increases up to 22 kHz, where the PR values reach a maximum and their corresponding dispersion is dramatically reduced, which is consistent with the occurrence of only fully extended wave functions. This is corroborated again by the corresponding values of *ξ*, which once more exceed by several orders of magnitude the actual size of the system as well as, contrary to the PR values, their dispersion.

A comparison between a TBT calculation of PR values and the experiment for the same realization {*n*_*i*_}, on the one hand, shows some significant deviations, however the numerical calculations are consistent with the experiment. On the other hand, a comparison between a TBT calculation of the length of localization *ξ* and the experiment is very good. In the inset of Fig. 5, we also show the participation ratio for the same ensemble, but for a disorder Δ = 1. It is observed that the gap at low frequencies disappear, the dispersion of the calculated PR values increases significantly below *f*_*c*_ while the average value of the PR decreases. This is consistent with a transition to a regime where the localization of the wave amplitudes may occur below *f*_*c*_. For normal-mode frequency values beyond 30 kHz but below *f*_*c*_, both PR and *ξ* average values decrease uniformly.

### Nearest-neighbor spacing distribution

**Figure 7:**
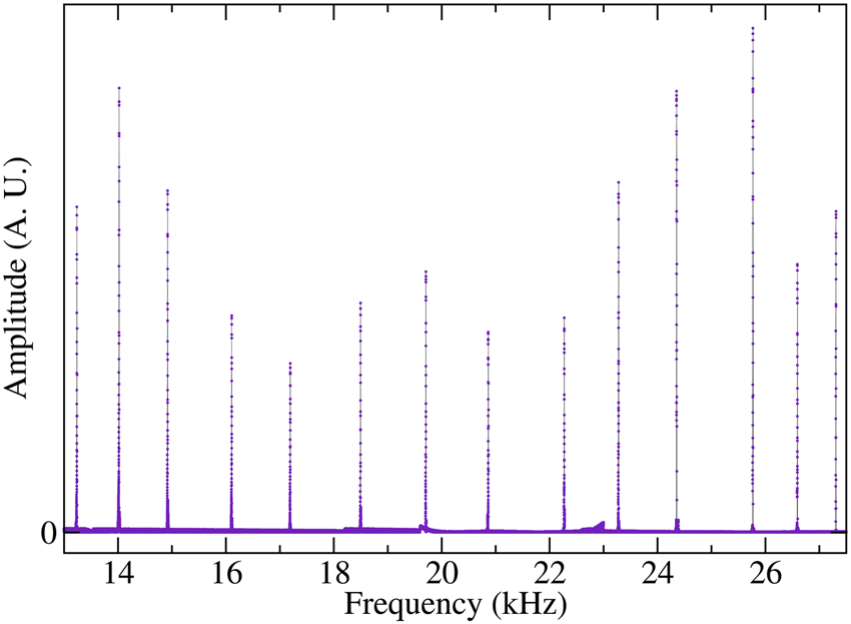
Measured spectrum for the system of Fig. 1. Notice that with the experimental configuration used, practically only flexural modes were excited in this interval of frequencies.

In obtaining the wave amplitudes, such as those shown in Fig. 3, the spectrum of the disordered beam must first be obtained. This is the case both numerically and experimentally. In Fig. 7 the spectrum measured for the system of Fig. 1, is shown. We are then provided with the statistical properties of the elastic spectra which render themselves to studies like those analyzed in spectral statistics and quantum chaos^36,37^. In what follows we will calculate the nearest-neighbor spacing distribution *p*(*s*_*j*_), where ${s}_{j}=\frac{{f}_{j+1}-{f}_{j}}{\langle {f}_{j+1}-{f}_{j}\rangle }$ is the normalized spacing, and show how the distribution changes as the frequency is increased. Before obtaining the nearest-neighbor spacing distribution, it is necessary to get rid of secular variations of the level density. We have therefore performed the procedure known as unfolding^36^.

**Figure 8:**
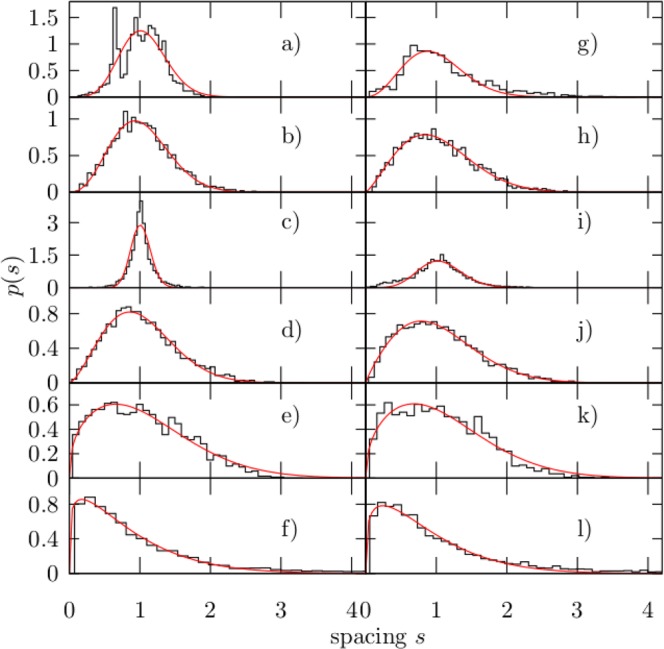
The nearest-neighbor spacing distribution histogram *p*(*s*) numerically obtained for an ensemble of 200 beams with a disorder Δ = 0.4 (left column) and averaged over a frequency interval as specified in Table 1. Figures (**a**–**f**) correspond to the intervals 1–6, respectively. The continuous (red) curves correspond to least-squares fittings to Eq. (1) and the corresponding obtained values for *α* in each case, are also shown in Table 1. The nearest-neighbor spacing distribution histogram *p*(*s*) obtained for the same ensemble of 200 beams but with a disorder Δ = 1 is shown in the right column (figures **g**–**l**) and the corresponding values of *α* are shown in the fourth column of Table 1.

We first consider an ensemble of 200 disordered beams from a numerical point of view. The distribution of energy levels in complex many particle quantum systems are surprisingly well described by the Gaussian orthogonal (GOE), the Gaussian unitary (GUE), and the Gaussian symplectic (GSE) random matrix ensembles^38,39^. A more general distribution that allows to study intermediate statistics between the ensembles just mentioned, is the one proposed by Izrailev^31^

1 $${p}_{\alpha }(s)=A{s}^{\alpha }{\mathrm{(1}+B\alpha s)}^{f(\alpha )}\exp [-\frac{{\pi }^{2}}{16}\alpha {s}^{2}-\frac{\pi }{2}\mathrm{(1}-\alpha \mathrm{/2)}s],$$

where

2 $$f(\alpha )=\frac{{2}^{\alpha }}{\alpha }\mathrm{(1}-\alpha \mathrm{/2)}-0.16874,$$

and *A*, *B* are normalizing constants determined by ${\int }_{0}^{\infty }\,p(s){\rm{d}}s=1$ and ${\int }_{0}^{\infty }\,sp(s){\rm{d}}s=1$. The parameter *α* is determined by fitting Eq. (1) to the numerical distribution by means of a least-squares fitting procedure. The distributions *p*_*α*_(*s*), numerically obtained for several regions of the frequency spectrum, are shown in Fig. 8. Unfortunately, the number of frequencies for a single beam is very small and the corresponding statistics is very poor both numerically and experimentally. It should be stressed, however, that the parameter *α* in Eq. (1) has to be considered as a one that describes globally the nearest-neighbor spacing distribution and not the level repulsion only^33^, since the small spacing behavior (*s* ≪ 1) in the Anderson model strongly depends on the specific properties of the system^40–42^. For *α* = 1, 2, 4 the parameter *α* coincides with the Dyson parameter *β*^43^. We remark here that the parameter *α* allow us to characterize the nearest-neighbor spacing distribution, however it is not related to any symmetry.

Different intervals in frequency were chosen according to the behaviour observed in the spectrum of Fig. 2, and from results of Figs 5 and 6. For zero or very small disorder, Δ ≪ 1, the spectrum should correspond to a periodic or quasi-periodic system, therefore the normal-mode frequencies must be equally spaced in each band. In this scenario, the nearest-neighbor spacing distribution should be of the form *p*(*s*) → *δ*(*s* − 1) and the normal-mode wave amplitudes should be fully extended. This situation is observed in Fig. 8 only for the third interval of frequencies below *f*_*c*_ and for Δ = 0.4, as defined in Table 1, which is also reflected in the relatively large values obtained for the parameter *α*, as shown in the third column of Table 1. This is in agreement with the large values of PR and the localization length *ξ* observed in the same interval of frequency in Figs 5(a) and 6(a). We emphasize that increasing the disorder up to Δ = 1, reduces the value of *α* for frequencies below *f*_*c*_, but it is still relatively large for the third interval of frequencies, as shown in the right column of Fig. 8 and in the fourth column of Table 1. This result suggest that a quasi-periodicity persists below *f*_*c*_ despite the disorder in the system is very high.

**Table 1:** Obtained *α* values as a function of the frequency.

Interval #	Freq. range (kHz)	*α* (Δ = 0.4)	*α* (Δ = 1)
1	0–3	4.2	2.26
2	3–17	2.4	1.18
3	17–34	23.29	4.82
4	34–50	1.41	0.82
5	50–60	0.38	0.4
6	60–70	0.2	0.26

Surprisingly, in the first and second interval of small frequencies, as defined in Table 1, the distribution *p*(*s*) for Δ = 0.4, is not a Dirac’s delta distribution, which suggest a weak response to the disorder from the states lying on the edge of residual bands, again in agreement with the results of Figs 5(b) and 6(b), and therefore consistent with the phenomenon of pre-localization^9^. For strong disorder, on the other hand, all eigenstates should be localized and the nearest-neighbor spacing distribution must be close to a Poissonian distribution *p*(*s*) → exp(−*s*), which is obtained from Eq. (1) in the limit *α* → 0. This situation is also observed in Fig. 8 for the fifth interval of frequencies and accentuated in the sixth interval. In this extreme case, the value obtained for the parameter *α* is close to zero, as shown in the fourth column of Table 1. Once again, this result is consistent with the results of Figs 3, 5 and 6.

In between Dirac and Poissonian distributions, we expect intermediate statistics which could be compared with the Wigner-Dyson distribution that emerge in classically chaotic systems described by full random matrices or in quasi-one-dimensional models described by banded random matrices^44,45^, when the localization length is larger than the sample size^33^. Thus, in the fourth interval of frequencies (see Table 1), a transition from fully extended to localized wave functions is expected and indeed observed, which rises the question about the relation between the localization length and the nearest-neighbor spacing distribution parameter *α* in the transition of these two limits. Unfortunately, in our case the density of states is not large enough to perform a statistical analysis with more resolution in frequency.

## Conclusion

In conclusion, in this letter we have presented, both numerically and experimentally, evidence of the Anderson localization in an elastic system under flexural oscillations. The localization of the normal-mode wave functions was experimentally observed by measuring the wave amplitude of an elastic beam and by calculating the participation ratio PR and the length of localization *ξ*. A good agreement between the numerical simulations and the experimental values was obtained. We have also calculated the parameter *α* describing the nearest-neighbor spacing distribution. We have found that the effect of the disorder is stronger above the crossover frequency *f*_*c*_ than below it, which is produced by a higher level density above *f*_*c*_. Completely localized or extended wave functions have been observed in the same system at relative high frequencies. The first ones, occur above *f*_*c*_ which is corroborated by the calculated values of PR, *ξ* and *α*, while the second ones occur below *f*_*c*_ at frequencies that are not small. It is worth to mention that our findings have been not observed in other systems.

## Methods

### The Timoshenko beam theory

The Timoshenko beam theory used to numerically study the Anderson phenomenon in flexural oscillations is based on the following equation, obeyed by the displacement *ζ* of the neutral axis^29^:

3 $$\frac{EI}{\rho S}\frac{{\partial }^{4}\zeta }{\partial {z}^{4}}-\frac{I}{S}(1+\frac{E}{\kappa G})\frac{{\partial }^{4}\zeta }{\partial {z}^{2}\partial {t}^{2}}+\frac{{\partial }^{2}\zeta }{\partial {t}^{2}}+\frac{\rho I}{\kappa GS}\frac{{\partial }^{4}\zeta }{\partial {t}^{4}}=0.$$

Here *E* and *G* are the Young and shear moduli, respectively, *ρ* is the mass density, *I* is the second moment of area and *S* is the cross section area of the beam. The only free parameter in this theory is the so called Timoshenko shear coefficient *κ*, which summarizes all the information about the distribution of shear stresses over the cross section of the beam. Analytic expressions of *κ* for different cross sections are available in the literature^46–49^, which are obtained under the common assumption that the cross section of the beam remains flat under flexural oscillations. This assumption however, is not always true, as demonstrated by experimental measurements^30,35^, specially at relatively high frequencies. Therefore, a dynamic coefficient *κ* is required in order to describe more accurately the bending oscillations of a beam like the one shown in Fig. 1.

Besides the transverse displacement in the *x*-axis direction *ζ*(*z*, *t*), Timoshenko introduced an angular variable *ψ*(*z*, *t*), which indicates the rotation of the cross section with respect to the *y*-axis. Variables *ζ* and *ψ* obey the following system of coupled equations:

4 $$\kappa GS(\frac{\partial \psi }{\partial z}+\frac{{\partial }^{2}\zeta }{\partial {z}^{2}})=\rho S\frac{{\partial }^{2}\zeta }{\partial {t}^{2}},$$

and

5 $$\kappa GS(\psi +\frac{\partial \zeta }{\partial z})-EI\frac{{\partial }^{2}\psi }{\partial {z}^{2}}=-\,\rho I\frac{{\partial }^{2}\psi }{\partial {t}^{2}}.$$

From these two equations, the fourth-order Timoshenko Eq. (3) for transverse displacement *ζ* of a uniform beam is obtained. An identical equation holds for angle *ψ*. Imposing normal-mode conditions *ζ*(*z*, *t*) = *χ*(*z*)*e*^*iωt*^ and *ψ*(*z*, *t*) = *φ*(*z*)*e*^*iωt*^, where *ω* = 2*πf*, *f* being the frequency, the Timoshenko equation admits the solution

6 $$\chi (z)=A{e}^{{k}_{1}z}+B{e}^{{k}_{2}z}+C{e}^{{k}_{3}z}+D{e}^{{k}_{4}z},$$

where the wavenumbers

7 $${k}_{m}={(-\mathrm{1)}}^{[m\mathrm{/2]}}\sqrt{-\alpha +{(-\mathrm{1)}}^{m}\sqrt{{\alpha }^{2}+4\beta }},\,m=1,\ldots ,4,$$

depend on the mechanical and geometrical properties of the beam through the definitions:

8 $$\alpha =\frac{\rho {\omega }^{2}}{2{M}_{r}},\beta =\frac{\rho {\omega }^{2}S}{EI}(1-\frac{{\omega }^{2}}{{\omega }_{c}^{2}}),\,{\rm{and}}\,{\omega }_{c}=\sqrt{\frac{\kappa GS}{\rho I}}.$$

Here $\frac{1}{{M}_{r}}=\frac{1}{E}+\frac{1}{\kappa G}$ and [*m*/2] is the largest integer less or equal than *m*/2. Note from Eqs (7) and (8) that the nature of the solutions changes drastically when *ω* crosses the crossover value *ω*_c_: below it, the first and third of the terms in Eq. (6) correspond to periodic oscillations while the other two, the second and fourth correspond to spatial exponential decay and growth, respectively. In this regime, only one wavelength appears, *λ*_1_ = 2*π*/|*k*_1_|, where |*k*_1_| denotes the amplitude of *k*_1_. For *ω* > *ω*_c_, however, the two terms corresponding to spatial exponential decay and growth become propagating periodic oscillations and therefore, a second wavelength appears, *λ*_2_ = 2*π*/|*k*_2_|.

The relative phase of the two wave-components appearing above *ω*_c_, becomes crucial in the dynamics of the vibrating beam, since they can interfere constructively or destructively. In ref.^50^ it was shown that constructive interference causes the warping of the end cross sections for half of the normal modes of a uniform free-free beam, while destructive interference results in flat end cross sections for the other half of normal modes^30^. These findings show that the assumption of flat cross sections, made in obtaining analytic expressions for *κ*, no longer holds above *ω*_c_. However, by means of a least-squares fitting procedure applied to the spectrum of frequencies, calculated using a three dimensional finite element method (FEM 3D), we obtain a simple relation for *κ* as a function of the frequency *ω*, which reproduces the normal-mode frequencies with an error smaller than 2%

9 $$\kappa (\omega )=\{\begin{array}{cc}{\kappa }_{0}+a\omega +b{\omega }^{2}, & \omega  < {\omega }_{0},\\ {\kappa }_{1}, & \omega  > {\omega }_{0},\end{array}$$

where *κ*_0_, *a*, *b* and *κ*_1_ are fitting parameters. Here, *ω*_0_ = 60 kHz.

For a structured beam like the one of Fig. 1, we introduce local coordinates, thus the amplitude *χ*_*j*_(*z*) in the *j*-th beam is given by

10 $${\chi }_{j}(z)={A}_{j}{e}^{{k}_{1,j}(z-{z}_{j})}+{B}_{j}{e}^{{k}_{2,j}(z-{z}_{j})}+{C}_{j}{e}^{{k}_{3,j}(z-{z}_{j})}+{D}_{j}{e}^{{k}_{4,j}(z-{z}_{j})},$$

and a similar expression for *φ*_*j*_(*z*). Here *z*_*j*_ ≤ *z* ≤ *z*_*j*+1_, *j* = 1, 2, .., *N*, and *k*_*m*, *j*_ with *m* = 1, .., 4 is given by an identical equation to Eq. (7), but now *S* and *I* in Eq. (8) should be replaced by *S*_*j*_ and *I*_*j*_, the cross sectional area and the second moment of area, respectively, of the *j*-th beam. Continuity conditions of amplitude functions *χ* and *φ*, bending moment

11 $$M(z)=-\,EI\frac{\partial \psi }{\partial z},$$

and shear force

12 $$Q(z)=-\,\kappa SG(\psi +\frac{\partial \zeta }{\partial z}),$$

at *z*_*j*+1_ (*j* = 1, 2, .., *N*) are then applied to Eq. (10), which allows the coefficients *A*_*j*+1_, *B*_*j*+1_, *C*_*j*+1_ and *D*_*j*+1_ to be expressed in terms of *A*_*j*_, *B*_*j*_, *C*_*j*_ and *D*_*j*_ as follows,

13 $${({A}_{j+1},{B}_{j+1},{C}_{j+1},{D}_{j+1})}^{T}={{\bf{M}}}_{j\to j+1}{({A}_{j},{B}_{j},{C}_{j},{D}_{j})}^{T},$$

where *T* means the transpose and **M**_*j*→*j*+1_ is a 4 × 4 diagonal matrix calculated as in ref.^34^. Applying successively **M**_*j*→*j*+1_ we obtain

14 $${({A}_{N},{B}_{N},{C}_{N},{D}_{N})}^{T}={{\bf{M}}}_{N-1\to N}{{\bf{M}}}_{N-2\to N-1}\cdot \cdot \cdot \cdot {{\bf{M}}}_{1\to 2}{({A}_{1},{B}_{1},{C}_{1},{D}_{1})}^{T}.$$

Vanishing of bending moment and shear force at *z* = *z*_1_ and *z* = *z*_*N*_ provide the boundary conditions. These lead to two algebraic equations for coefficients *A*_1_, *B*_1_, *C*_1_, *D*_1_ and two more for coefficients *A*_*N*_, *B*_*N*_, *C*_*N*_, *D*_*N*_. If this last set of coefficients is eliminated using Eq. (14) we obtain a homogeneous system of algebraic equations in the four unknowns *A*_1_, *B*_1_, *C*_1_, *D*_1_. The determinant of such algebraic system must vanish to obtain the normal-mode frequencies.

### Experimental measurements

Experimental measurements were performed using the electromagnetic-acoustic transducer (EMAT) described in ref.^51^. The EMAT can be used either to detect or excite oscillations and according to the relative position of the magnet and the coil of the EMAT, it can either excite or detect selectively compressional, torsional or flexural vibrations. This transducer has the advantage of operating without mechanical contact with the beam, which turns out crucial to avoid perturbing the shape of the localized wave amplitudes. Both the detector and exciter can be moved automatically along the beam axis and then the wave amplitudes can be measured easily. However, a process of elimination of modes corresponding to compressional and torsional vibrations is still required^35^. This procedure may become quite tedious and is the reason that only a few normal-mode wave functions were obtained in the experiment. In Fig. 1 we show the general setup used to excite and detect flexural waves. A signal generator sends a sinusoidal signal to the power and lock-in amplifiers, which serves as a reference signal for the latter. The power amplifier sends the amplified signal to the exciter. Then, the detector sends the signal to the lock-in amplifier which is responsible for the filtering of the signal. At the end, the signal is sent back to the digital-to-analog converter (DAC) and then to the computer, which records the data.

### Inverse participation ratio and localization length

For a given normal-mode wave function *χ* the IPR is given by

15 $${\rm{IPR}}=\frac{{\Sigma }_{j}{\chi }_{j}^{4}}{{({\Sigma }_{j}{\chi }_{j}^{2})}^{2}},$$

which on the average measures the number of sites that contribute significantly to the wave function. For localized states, the IPR is directly connected with the localization length *ξ*^52^, which measures how the wave amplitudes of the normal modes decay exponentially and is defined trough the amplitude envelope of the wave function *χ*

16 $${\chi }_{{\rm{env}}}={\chi }_{0}\,\exp \,(-\frac{|x-{x}^{\ast }|}{\xi }),$$

where *χ*_0_ is a constant and x^*^ is the position of the maximum of the wave amplitude. Notice that in this letter we use the PR instead of its reciprocal, the IPR, for reasons of convenience.
